# Detection of *Brucella* S2 vaccine strain by a loop-mediated isothermal amplification (LAMP) method

**DOI:** 10.3389/fcimb.2022.1023243

**Published:** 2022-11-28

**Authors:** Jiaming Mu, Qi Li, Xu Yan, Xiaowei Mao, Yaqin Shi, Yun Qin, Chunxia Liu, Wenlong Wang

**Affiliations:** ^1^ College of Veterinary Medicine, Inner Mongolia Agricultural University, Huhehot, China; ^2^ College of Life Science, Inner Mongolia Agricultural University, Huhehot, China

**Keywords:** Brucellosis, *Brucella* spp., LAMP assay, Brucella S2 vaccine, differential diagnosis

## Abstract

**Introduction:**

Brucellosis is a highly prevalent zoonotic disease caused by *Brucella* spp. *Brucella suis* S2 vaccination is an effective strategy to prevent animal brucellosis. However, S2 induces antibodies against the smooth lipopolysaccharide,making it challenging to distinguish field infected from vaccinated livestock. Early and accurate diagnosis is essential for infection control and prevention. In this study, we aimed to develop a quick and accurate assay to distinguish the *BrucellaS2* vaccine strain from closely related *B. abortus* and *B. melitensis*.

**Methods:**

Whole-genome sequencing of *B. suis S2* was performed, and the sequence was compared with that of the genomes of *B. abortus* and *B. melitensis*. One specific gene, *GL_0002189*, was selected as a marker to differentiate the *Brucella*S2vaccine strain from *B. abortus* and *B. melitensis*. A loop-mediated isothermal amplification (LAMP) assay was developed, based on the *GL_0002189* gene, and then assessed for target specificity, lower limit of detection, and repeatability.

**Results:**

Our results revealed that there was no cross-reaction with other strains, and the LAMP assay displayed high sensitivity for detecting S2 with a minimum detection limit of 18.9×103 copies/µL DNA input, it is nearly 100 times higher than conventional PCR technology. Concordance between the LAMP assay and a conventional polymerase chain reaction method was assessed using 54 blood samples collected from sheep with suspected brucellosis. Total concordance between the two assays was 92.6%, without a significant difference (p > 0.05) in the test results.

**Conclusion:**

This is the first report of a LAMP assay for the detection of the *B. suis* S2vaccine strain. Our approach can be helpful for the control and eradication of brucellosis, and its simplicity in requiring no specialized equipment or personnel makes it useful for implementation in resource-limited settings as well as for field use.

## Introduction

Brucellosis, caused by *Brucella* spp., is a serious zoonotic disease, which is prevalent worldwide (Kamga et al., 2020). It causes serious economic loses for farmers through the reduction of productivity (El-Sayed and Awad, 2018). The clinical symptoms caused by brucellosis include abortion, infertility, decreased production, and undulating fever with arthralgia in humans (Cao et al., 2018). In some economically developed countries, brucellosis in domestic animals is mainly purified by detection and positive killing to achieve prevention and control. In China, in recent years, brucellosis in cattle, sheep, and humans has increased to varying degrees since low prevalence areas do not carry out vaccine immunization, and the positive animals are eliminated to purify the herds. In areas with severe numbers of brucellosis cases, the infection in cattle and sheep can be prevented and controlled by vaccination. According to the survey, cattle, sheep and caprine brucellosis is predominantly related to *B. melitensis*, as well as *B. abortus* (Dadar and Alamian, 2021). The live attenuated vaccine strains play an important role in the prevention and control of animal and human brucellosis (Goodwin and Pascual, 2016). Although attenuated vaccines do not completely protect against *Brucella* spp. infection, vaccination is currently the most effective prevention and control strategy against brucellosis (Wang et al., 2020). In China, live attenuated *Brucella suis* vaccine (*Brucella suis* S2) is the most widely used brucellosis vaccine. The *Brucella* S2 vaccine is quite effective in preventing livestock brucellosis, such as in sheep, goats, cattle and pigs (Zhu et al., 2016). It has many advantages, including high safety, efficacy, and multiple vaccination routes. Nevertheless, S2 induces antibodies against the smooth lipopolysaccharide, making it challenging to distinguish field infected from vaccinated sheep, cattle and pigs.

Diagnosis of brucellosis is mainly based on bacteriological and immunological tests, which include Rose-Bengal plate agglutination test, standard tube agglutination test, Fluorescence polarization assay, complement fixation test, and enzyme-linked immunosorbent assay (Ducrotoy et al., 2018). Therefore, under the reality of vaccination against brucellosis, it is urgent to develop a differential diagnosis approach for commonly used vaccines. The advantages of Loop Mediated Isothermal Amplification (LAMP) analysis are that it is easy to operate, requires less special equipment, has high accuracy, can be directly interpreted by naked eye, and can be used in places with limited instrument resources (Trangoni et al., 2015). The colorimetric LAMP assay is rapid and can be used in the field, such as farm sites, as it does not require specialized equipment and personnel (Sathish Kumar et al., 2021).

The qPCR method was used to detect the target nucleic acid in the peripheral blood of 54 sheep. The target gene encoded the *Brucella* ABC transporter permease (NCBI Reference Sequence: WP_002965788.1), which is a common gene of the *Brucella* wild strain and *B.suis* S2 vaccine strain. The *B.suis* S2 vaccine is a live attenuated vaccine, after inoculation, nucleic acid of the *B.suis* S2 vaccine strain can be detected. Therefore, qPCR test results cannot determine whether 54 sheep are infected with the *Brucella* wild strain or *B.suis* S2 attenuated vaccine, and further differentiation is needed. In this study, comparative genomics research methods were used to sequence the whole genome of the widely used *Brucella* S2 vaccine in China, analyze and compare it with the genomes of six classical species of *Brucella* published by GenBank, and screen out several differential genes, including *GL_0002189* which was selected as a marker gene to distinguish *Brucella* S2 vaccine from *Brucella melitensis* and *Brucella abortus*. Therefore, this study is based on the LAMP detection method using *GL_0002189* marker gene for the distinguishing of *Brucella* S2 vaccine strain from the aforementioned strains. In addition, we evaluated the specificity, lower detection limit and repeatability of LAMP (*GL_0002189*). Lastly, we planned to compare concordance between the LAMP assay and a conventional PCR-based approach in order to investigate the clinical utility of the LAMP assay in low-resource settings as well as for field use. Our methods help prevent and control brucellosis.

## Methods and materials

### Bacteria strains and DNA

*B. abortus* S19, *B. abortus* A19, *B. melitensis* Rev.1, *B. melitensis* M28, and *B. suis* S2 were tested in this study along with three non-*Brucella* species, namely *Streptococcus*, *Salmonella*, and *Staphylococcus aureus*. Genomic DNA was extracted from the isolates using the Genomic Template Extraction Kit (Tian gen Biotech Co. Ltd, Beijing, China), according to the manufacturer’s protocol, and quantified using Nanodrop (Gene Company Limited).

### Comparative genomics research

Whole-genome sequencing for *B. suis S2* was performed using Illumina HiSeq 2000. A comparative genomics investigation was carried out with the whole-genome sequences of the field epidemic strains, *B. melitensis* and *B. abortus*, using the Basic Local Alignment Search Tool (BLAST) of the National Center for Biotechnology Information (NCBI) (https://blast.ncbi.nlm.nih.gov/Blast.cgi). The essential data of *Brucella* spp. are shown in **Table 1**.

**Table 1:** The basic information of *Brucella* spp.

Species	Strains	GenBank login number	Chromosome	Size	GC%	Gene	Protein
*B.abortus*	9-941	AE017224.1	2	3.29	57.2	3355	3084
*B.abortus*	2308	AM040265.1	2	3.28	57.2	3418	3034
*B.abortus*	S19	CP000888.1	2	3.28	57.2	3133	3000
*B.melitensis*	M28	CP002460.1	2	3.31	57.2	3427	3363
*B.melitensis*	M5-90	CP001852.1	2	3.31	57.2	3421	3357
*B.melitensis*	NI	CP002932.1	2	3.29	57.2	3290	3229
*B.suis*	S2	CP045138.1	2	3.33	57.2	3243	3243

### Percentage of identity comparison and PCR detection

A BLAST alignment of the target gene was performed against *Brucella* spp. genomes published on the NCBI database to investigate percentage of identity. Simultaneously, designed PCR primers for target gene. The forward and backward primers utilized were 5′-CCTACAACCCATCGAAGTGG-3′ and 5′-TCTTGAGCAAGAACCAGCAC-3′, respectively. The one-step assay, which takes approximately 3 h to complete, was composed of one denaturing cycle at 95°C for 5 min, followed by 30 cycles at 94°C for 45 s, 56°C for 45 s, and 72°C for 45 s, and the final extension step was performed at 72°C for 10 min. The PCR amplification products were analyzed using electrophoresis on 1% agarose gel. The reaction components for the PCR included 12.5 μL Premix Taq (Takara Biomedical Technology Co., Ltd., Beijing, China), 1μL of forward and backward primers (final concentration was 10 µmol/L), 1 μL of the DNA template, and then made up to 25 μL with DNase/RNase-free water.

### LAMP primers design

The LAMP primers based on the target gene were designed using Primer Explorer V5 software (http://primerexplorer.jp/e/). The primer information is shown in **Table 2**.

**Table 2:** The LAMP primers information.

Primers	Sequence information(5′-3′)
F3	CCTACAACCCATCGAAGTGG
B3	TCTTGAGCAAGAACCAGCAC
FIP	ACCGAACACGCCAGCCAAGTGCTTGTGTTCCTGCG
BIP	TAAGTCCGGGCGCTCACCATGGAGCGCGAATGAGC
LF	CCTCCTCGACCGTGATCT
LB	ATCAGATGACGCGGCTTG

### Optimization of LAMP assays

The LAMP assay was optimized in a 25 μL reaction system, which included MgSO_4_ (New England Biolabs Co., Ltd, Beijing, China), dNTPs (Takara Biomedical Technology Co., Ltd., Beijing, China), and LAMP primers (Sangon Biotech Shanghai Co., Ltd., Shanghai, China). To improve the LAMP conditions, the reactions were assessed at various concentrations of MgSO_4_ (2–10 mmol/L), dNTPs (0.6–3.0 mmol/L), outer primers F3/B3 (0.1–0.5 µmol/L), inner primers FIP/BIP (0.8–2.8 µmol/L), loop primers LF/LB (0.1–1.0 µmol/L), and reaction temperatures (59–69°C). End-point analysis of the products of the assay was performed using agarose gel electrophoresis and a visual test, using SYBR Green I dye (Beijing Solarbio Science & Technology, Beijing, China). For the visual test, under visible light, fluorescent green indicated the presence of the amplicon, while orange indicated.

### Specificity of the LAMP assay

The specificity of the LAMP assay target gene, was analyzed using 10 ng/µL genomic DNA from the five *Brucella* strains and three non-*Brucella* strains. A 1 μL aliquot of genomic DNA was used as a template for LAMP reactions, and each sample was tested in triplicate.

### Sensitivity of the LAMP assay

Following the optimization of the LAMP assay, the sensitivity of the test was analyzed using 10-fold serial dilutions of 18.9×10^10^ copies/µL *B. suis* S2 DNA. For comparison, the 10-fold dilutions were further tested using PCR and the sensitivities of the two assays were compared.

### Repeatability of the LAMP assay

The repeatability of the LAMP assay was assessed by repeating the experiment using three different batches of reagents and following the optimized reaction conditions. The main components of the three batches of reagents are identical, but they are produced at different times to test the stability of this method in different batches of reagents. Each sample was tested in triplicate.

### Analysis of clinical samples

Fifty-four blood samples were collected from sheep that were suspected of being infected with wild strains in April 2022, the samples were stored in EDTA anticoagulation tubes. The sheep had been vaccinated with the *Brucella* S2 vaccine the previous year. DNA was extracted from the blood samples using the Genomic Template Extraction Kit (Tian gen Biotech Co. Ltd), according to the manufacturer’s protocol. The initial testing was performed using quantitative PCR, which confirmed *Brucella* spp. infection in 52 of the samples. However, the qPCR target gene encoded the *Brucella* ABC transporter permease (NCBI Reference Sequence: WP_002965788.1), which is a common gene of the *Brucella* wild strain and *B.suis* S2 vaccine strain. Therefore, qPCR test results cannot determine whether 54 sheep are infected with the *Brucella* wild strain or *B.suis* S2 attenuated vaccine, and further differentiation is needed. Using this gene as a molecular detection target, the nucleic acid of the *B. suis* S2 vaccine strain was positively detected, while that of *B. melitensis*, *B. abortus*, and other control strains were negatively detected. Therefore, all 54 samples were tested using the LAMP and conventional PCR methods established in this study to distinguish between the strains, so as to assess the clinical practicability of the LAMP assay as a diagnostic tool. The animal study was reviewed and approved by the Animal Ethics and Welfare Committee of Inner Mongolia Agricultural University.

## Results and analysis

### Results of comparative genomics

The genome of *Brucella* S2 vaccine strain was compared with that of six strains of *B. abortus* and *B. melitensis*. The results revealed a total of 20 *B. suis* S2-specific genes (**Table 3**), of which 7 genes with functional annotation were as follows: arginine ABC transporter permeability enzyme gene (*GL000288*), carmine transport permeability enzyme gene (*GL000289*), DNA replication and recombination gene (*GL002175*), phage gene (*GL002176*), protein binding gene (*GL002179*), type IV binding protein gene (*GL002181*), and endonuclease gene (*GL002186*). The remaining 13 genes are not functionally annotated. The *GL_0002189* gene was viewed as a marker that can differentiate the *Brucella* S2 vaccine strain from *B. abortus* and *B. melitensis*. Analysis indicates that there are some genome-level differences among the different genotypes of *Brucella*.

**Table 3:** Information of *Brucella suis* specific genes.

*Brucella suis* specific genes				
GL00288	GL00289	GL002175	GL002176	GL002177
GL002178	GL002179	GL002180	GL002181	GL002182
GL002183	GL002184	GL002185	GL002186	GL002187
GL002188	GL002190	GL002191	GL002192	GL0002189

### *GL_0002189* gene percentage of identity comparison and PCR detection results

**Figure 1:**
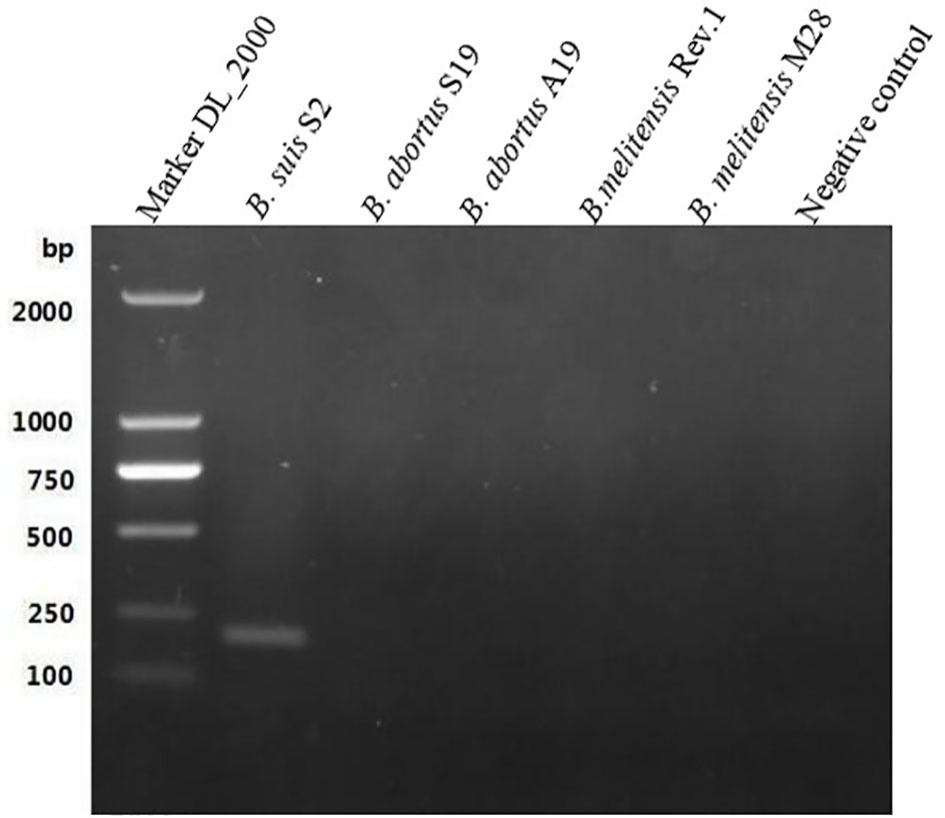
Polymerase chain reaction results of the GL_0002/89 gene. It was fotmd that the expected sizes (201 bp) were amplified only in the S2 vaccine strain, but not amplified in the *B. abortus* and *B.melitensis* strains tested in this study.

The length of the *GL_0002189* gene was 660 bp and the molecular weight was 24.1 kDa. The gene contained a 660bp complete open reading frame, encoding 219 amino acids. The protein encoded by this gene is hydrophilic, lacks transmembrane region, and is located in the outer bacterial membrane (https://trace.ncbi.nlm.nih.gov/Traces/index.html?view=study&acc=SRP394076). Based on the BLAST analysis, the *GL_0002189* gene sequence showed high percentage of identity with *B. suis*, *B. canis*, and *B. microti*, but it was not homologous with *B. abortus*, *B. melitensis*, and *B. ovis* (**Table 4**). The PCR amplification products were analyzed using electrophoresis on 1% agarose gel. It was found that the expected sizes (201 bp) were amplified only in the S2 vaccine strain, but not amplified in the *B. abortus* and *B. melitensis* strains tested in this study (**Figure 1**).

**Table 4:** BLAST analysis of the *GL_0002189* gene.

Strain name	Species	GenBank login number	Homology
*Brucella suis* bv.1 str. S2	*B.suis*	CP045138.1	100%
*Brucella melitensis* M5-90	*B.melitensis*	CP001852.1	0%
*Brucella abortus* bv.1 str.941	*B.abortus*	AE017224.1	0%
*Brucella ovis* ATCC 25840	*B.ovis*	CP000708.1	0%
*Brucella canis* strain RM6/66	*B.cains*	CP007759.1	99%
*Brucella microti* CCM 4915	*B.microti*	CP001579.1	99%

### Optimization of LAMP assay

Single-factor optimization of the LAMP reaction conditions was carried out for MgSO_4_, dNTPs, outer primers (F3/B3), inner primers (FIP/BIP), loop primers (LF/LB), and reaction temperature. Based on gel electrophoresis results, the amplicons that had the brightest bands on the agarose gel were produced using MgSO_4_ at 4 mmol/L, dNTPs at 1.8 mmol/L, F3/B3 at 0.2 µmol/L, FIP/BIP at 2.0 µmol/L, and LF/LB at 0.6 µmol/L. Therefore, these were considered optimal reaction conditions for the assay. Furthermore, while amplification was observed from 63–69°C, 65°C was chosen as the optimum temperature for the LAMP assay.

### Specificity of LAMP assay

**Figure 2:**
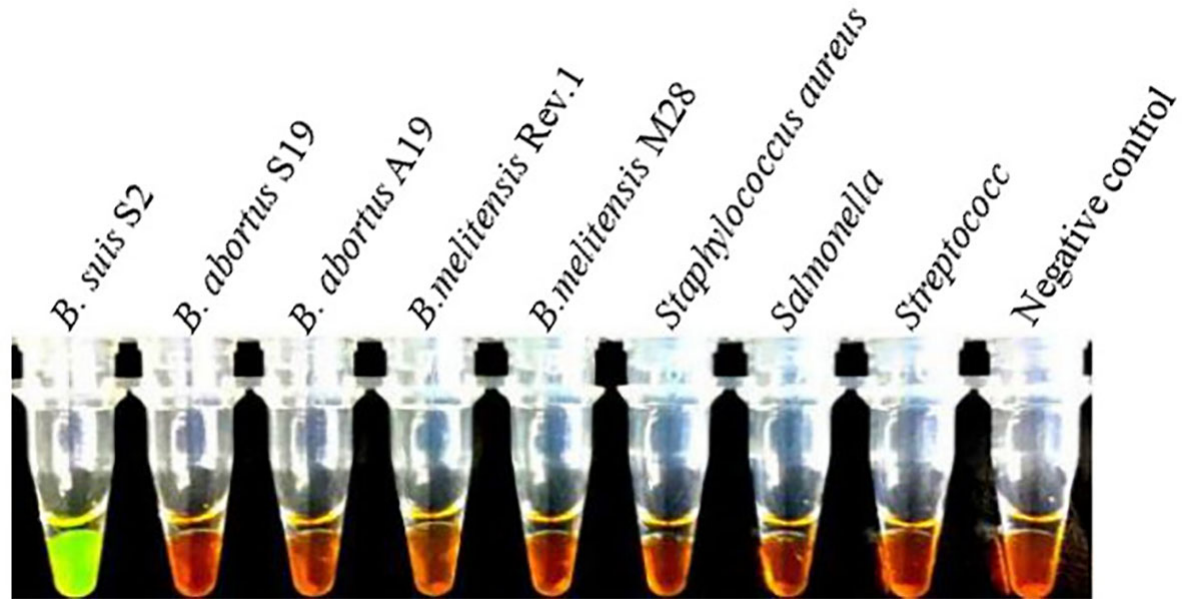
Target specificity of the LAMP assay. Fluorescent green indicates presence of the amplicon while orange indicates no amplification. Cross reaction was not observed when 10 ng/µL of DNA from *B. suis* S2, *B. abortus* Sl9, *B.abortus* Al9, *B.melitensis* Rev.1, *B.melite11sis* M28 strains, *Staphylococcus aureus*, *Salmonella*, *Streptococcus* DNA and negative control were used.

**Figure 3:**
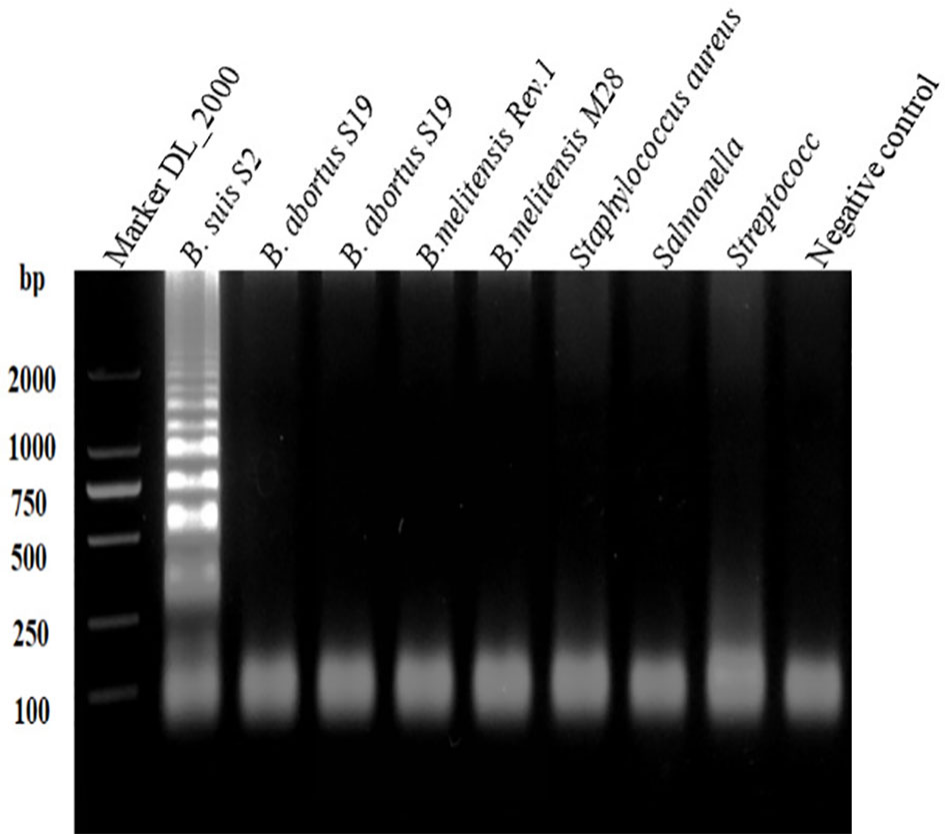
Target specificity of the L-\MP assay. Assay products were visualized using gel electrophoresis. Cross reaction was not observed when 10 ng/µL of DNA from *B. suis* S2, *B. abortus* SI9, *B. abortus* A19, *B.melitensis* Rev.1, *B.melitensis* M28 strains, *Staphylococcus aureus, Salmonella, Streptococcus* DNA and negative control were used.

**Figure 2** shows the visual assay used to determine the target specificity of the LAMP assay. Amplification was detected only for the *B. suis* S2 vaccine strain, indicated in fluorescent green, whereas no amplification was noted for the *B. abortus* and *B. melitensis* strains as well as the other three non-*Brucella* spp., indicated in orange. The results were also confirmed using agarose gel electrophoresis (**Figure 3**).

### Sensitivity of LAMP assay

**Figure 4:**
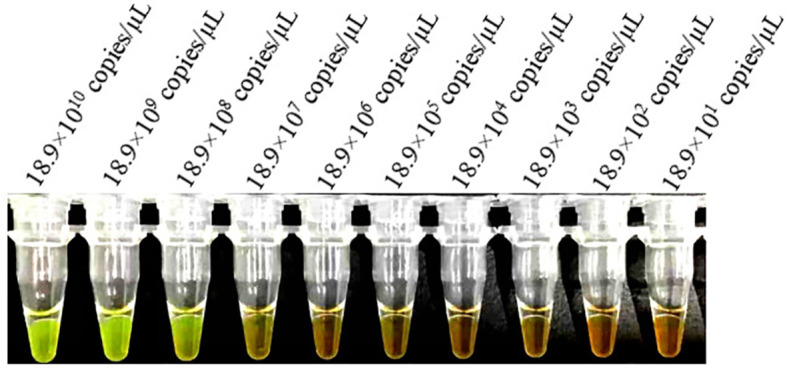
Lower limit of detect ion of the LAMP assay for the detection of *Brucella suis* S2 vaccine strain. Assay products were visualized using color change to detect different DNA concentrations. Fluorescent green indicates presence of the amplicon while orange indicates no amplification.

**Figure 5:**
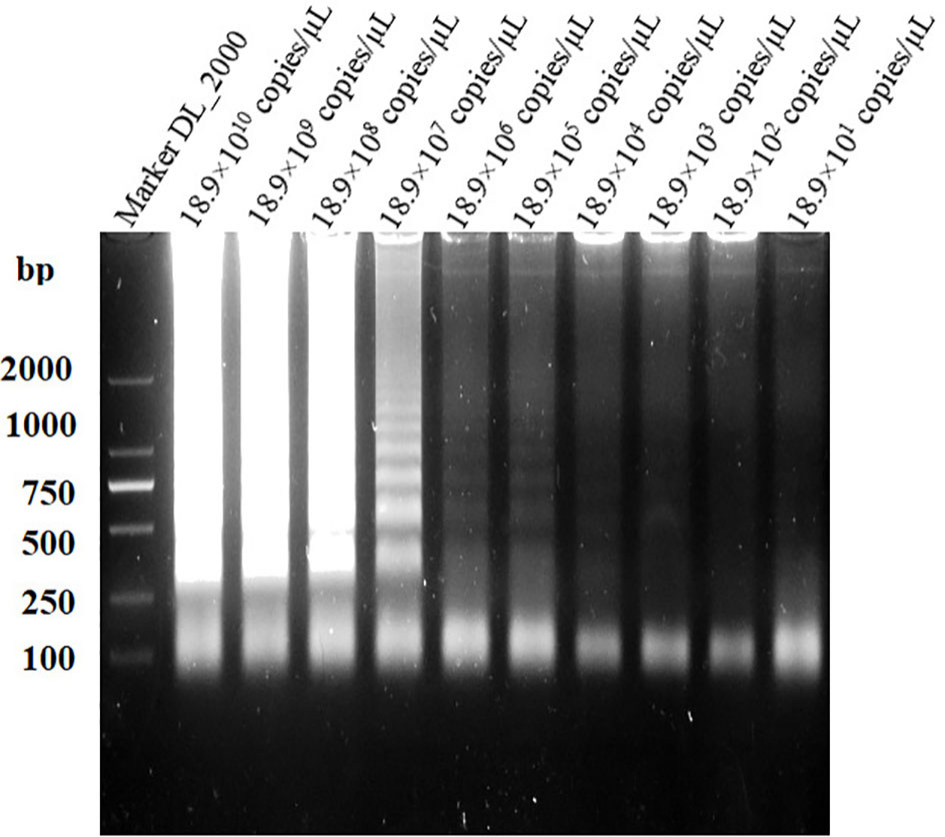
Lower limit of detection of the LAMP assay for the detect ion of *Brucella suis* S2 vaccine strain. Assay products were visualized using gel electrophoresis.

**Figure 6:**
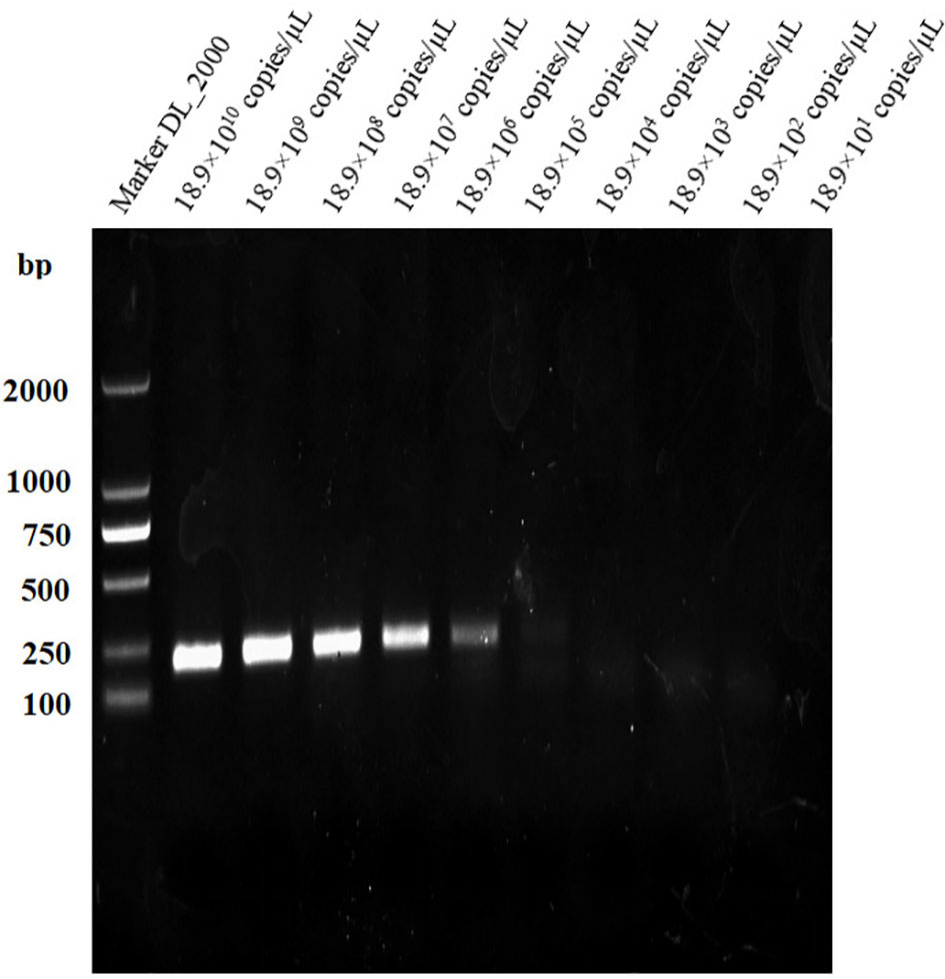
Lower limit of detection of the PCR assay for the detection of *Brucella suis* S2 vaccine strain. Assay products were visualized using gel electrophoresis.

The sensitivities of the LAMP and conventional PCR assays were evaluated and compared using serially diluted (10-fold) *B. suis* S2 DNA. The LAMP assay could detect as low as 18.9×10 (Goodwin and Pascual, 2016) copies/µL and 18.9×10^3^ copies/µL of S2 genomic DNA based on the visual and electrophoretic methods (**Figures 4**, **5**). Similarly, the conventional PCR assay could detect as low as 18.9×10^5^ copies/µL, based on agarose gel analysis (**Figure 6**). Therefore, the LAMP method is nearly 100 times higher than conventional PCR technology.

### Repeatability of LAMP assay

**Figure 7:**
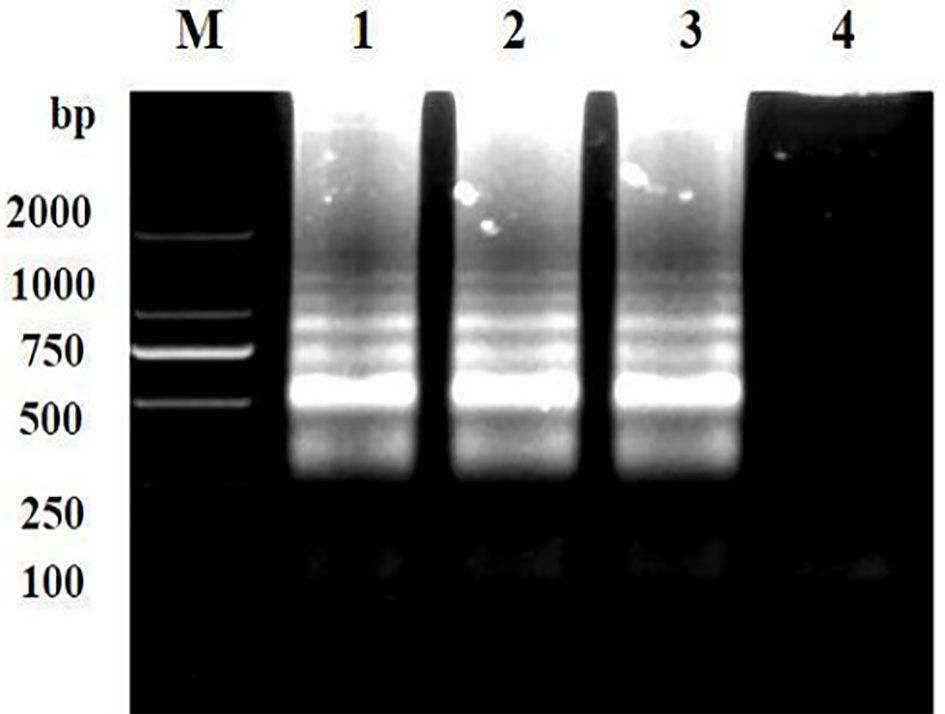
Repeatability detection of the LAMP assay for the detection of *Brucella suis* 52 vaccine strain. Assay products were visualized using gel electrophoresis. M, DNA marker DlL_2000; Lanes 1-3 are different batches of reagents and Lanes 4 is a negative control. The main components of the three batches of reagents are identical, but they are produced at different times.

**Figure 8:**
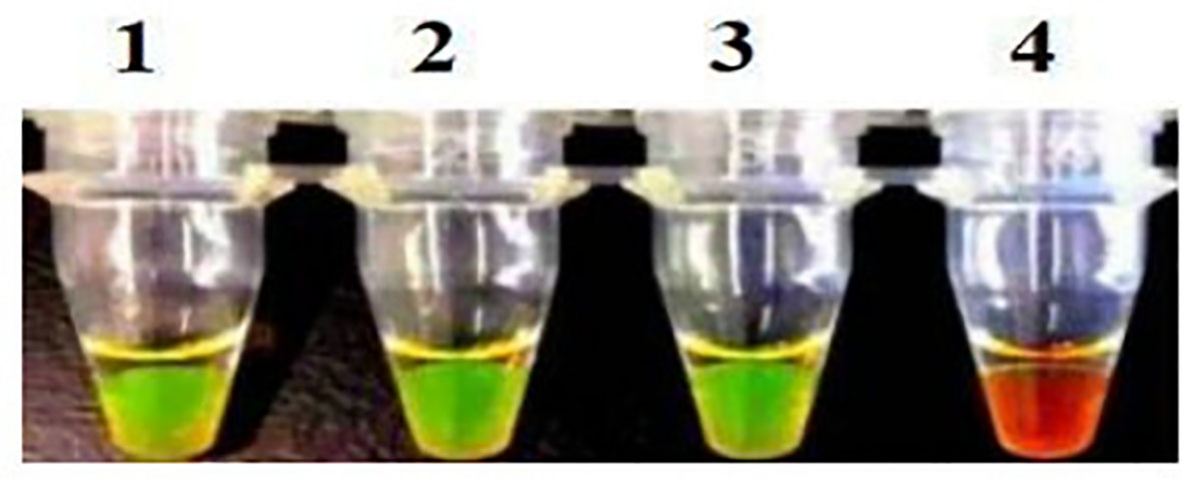
Repeatability detection of the LAMP assay for the detection of *Brucella suis* 52 vaccine strain. Assay products were visualized using color change. Pipe 1-3, three different batches of reagents. Fluorescent green indicates presence of the amplicon while orange indicates no amplification. The main components of the three batches of reagents are identical, but they are produced at different times.

Repeatability of the LAMP assay was assessed by repeating the experiment using different batches of reagents. Based on the visual and electrophoretic methods, amplification was detected after using the three distinct batches of reagents, indicating the repeatability of the LAMP assay (**Figures 7**, **8** **).**

### Clinical samples

The information provided in **Table 5** compares detection of *B.suis* S2 vaccine strain reliability of the established LAMP method targeting the *GL_0002189* gene with the conventional PCR method. The results showed *B.suis* S2 36 positive samples and 18 negative samples were successfully detected by LAMP, whereas *B.suis* S2 34 positive samples and 20 negative samples were successfully detected by conventional PCR. The positive coincidence rate of the two detection methods was 94.4% (34/36), the negative coincidence rate was 88.9%, and the total coincidence rate was 92.6%. There was no significant difference between the detection performance of the two methods, indicating that the LAMP method established in this study can be used to distinguish the *B.suis* S2 vaccine strain. The other 18 negative samples may be *B.suis* S2 vaccine strain or wild strains because the concentration of nucleic acid in their blood is lower than the detection limit of LAMP method. Therefore, to determine whether the 18 sheep were infected with the *B.suis* S2 vaccine strain or wild *Brucella* strains, a more sensitive detection method is needed.

**Table 5:** Clinical sample test results by PCR and LAMP methods.

Comparative method (PCR)	Test method (LAMP)		Total
	Positive	Negative	54
Positive	34	2	36
Negative	2	16	18

Overall percent agreement (OPA) = 100% × (34 + 16)/(34 + 2+2+16), Positive percent agreement (PPA) = 100% × 34/(34 + 2), Negative percent agreement (NPA) = 100% × 16/(2 + 16).

## Discussion

Brucellosis is a highly prevalent infectious disease and poses a major public health threat worldwide (Saber Marouf et al., 2021). The disease is caused by *Brucella* spp. and can cause multiple organ damage in humans and animals with serious health complications (Elfaki et al., 2015; López-Santiago et al., 2019). Vaccination of animals against the disease, using the *B. suis* S2 live-attenuated vaccine, has played an important role in reducing the incidence of brucellosis in humans and animals in the past decades (Masjedian Jezi et al., 2019). An effective way to eliminate brucellosis in livestock is to timely diagnose, identify, and eliminate brucellosis positive cattle and sheep. In countries where vaccination is widely used to prevent and control brucellosis, an effective way to eliminate brucellosis in herds in a short time period is to adopt detection methods that can diagnose and identify brucellosis natural infection and the immune response of animals to the vaccine. However, it can be a challenge to distinguish field infected from vaccinated livestock using current laboratory methods (Wang et al., 2014).

For nearly half a century, many economically developed countries have adopted quarantine and culling to purify livestock, which has achieved the control and eradication of brucellosis. China has adopted regional prevention and control approach. In areas where brucellosis is seriously prevalent, the method of detection and killing of positive animals was also used. However, the continuous emergence of positive animals has led to the necessity of preventing and controlling brucellosis infection in cattle and sheep through vaccination. Infection can be latent, and if field infected livestock are not culled from production, they can act as reservoirs of the pathogen for future infections. Thus, the ability to differentiate between wild type and vaccine strains is essential for the implementation of effective infection control and prevention protocols.

Based on some conserved genes and antigens of *Brucella*, such as *OMP25*, *BP26*, *BCSP31*, it is not possible to distinguish *B.suis* S2 vaccine strains from wild strains using fluorescence polarization and ultrasensitive detection technologies such as qPCR. However, few studies have been conducted to identify genes that differ between vaccine strains and wild strains. The target gene *GL_0002189* used in this study was screened by comparing the whole genome of *B.suis* S2 vaccine with that of the most prevalent *B. melitensis* and *B. abortus* strains. Therefore, using *GL_0002189* gene knot combined with qPCR, or using this gene to prepare a standard antigen combined with fluorescence polarization, could achieve the goal of ultrasensitive differentiation between vaccine strains and wild strains. Therefore, this study emphasizes the specificity of the *GL_0002189* gene, which could provide an important molecular target for many ultrasensitive diagnostic techniques. Fluorescence polarization, qPCR, ELISA, and other methods have shown excellent performance in disease detection, such as strong specificity and high sensitivity, but the instruments and equipment are generally expensive. In China and many developing countries, there are many small farms that do not have enough cost to buy instruments and equipment. The reason why LAMP technology was selected in this study is that it has low requirements for test instruments. Only a constant temperature device is needed, and the test results can be interpreted with the naked eye. This is a very convenient and low-cost detection method, the loop-mediated isothermal amplification strategy could be more widely used in regions with limited resources. However, this technology has some limitations. For example, the detection results are easily affected by aerosols due to the high sensitivity of the test (Notomi et al., 2015).

Investigation shows that *B. melitensis* and *B. abortus* are the main pathogens of *Brucella* infection in sheep, goats and cattle. Therefore, we analyzed the whole genome sequence of S2, compared it with the genome of *B. melitensis* and *B. abortus*, and screened some differential genes between the strain contained in the S2 vaccine natural virulent strains of *B. melitensis* and *B. abortus*. S2 vaccine specific *GL_ 0002189* gene research LAMP detection method was used to distinguish *B. melitensis*, *B. abortus*, and S2 vaccine strains. However, this method still has several limitations. For example, it cannot detect *B. suis* field strain infections, although a large number of studies have reported that livestock are mainly infected with *B. melitensis* and *B. abortus*.

As shown in the results of this study, 36 out of 54 clinical samples were detected as the *B.suis* S2 vaccine strain. The other 18 negative samples may be negative because the concentration of nucleic acid in their blood is lower than the detection limit of this method. Therefore, to determine whether the 18 sheep were infected with the *B. suis* S2 vaccine strain or other *Brucella* strains, a more sensitive detection method is needed. Studies by Cheville NF and others have shown that in goats inoculated with *B. Melitensis* 16M in the first 4 days of infection, the concentration of *Brucella* in milk was 10^4–^10^7^ CFU/mL. Furthermore, a bacterial culture of the superficial cervical lymph nodes produced a large number of bacteria in post-inoculation day 3 (PID3). Finally, at PID 7, the concentration of bacterial culture remained at a high level, but decreased significantly at 14-28 days. Bacteria can be isolated from blood in PID 1-2. Bacteriemia was detected in PID 3, but not in PID 116 (Cheville et al., 1996). In a study by Araya LN et al., BALB/c mice were infected intravenously with *B. abortus* strain 19. Subsequently, bacteria proliferated in large numbers in the spleen, the concentration reached a peak 2 weeks after infection (pi), and then the body gradually cleared the infection. After 8 weeks, the number of bacteria decreased by >10,000-fold (Araya et al., 1989). S I Muhammed et al. found that 20 ewes were inoculated with *Brucella ovis* on the vaginal mucosa from 5 to 40 days of pregnancy. Subsequent studies found that bacteremia was temporary or intermittent. After 98 days of infection, *Brucella ovis* could not be cultured from the blood or other tissues (Muhammed et al., 1975). It is not clear how long the *B.susi* S2 vaccine strain can exist in the blood after vaccination because it has not been studied. However, according to our research results, nucleic acids of the *B. suis* S2 vaccine strain can still be detected one year after vaccination.

In conclusion, we have found some differential genes that can distinguish *B. suis* S2 from *B. melitensis* and *B. abortus* through comparative genomics. Based on one of the genes (*GL_0002189* gene), LAMP detection method, based on a marker gene (*GL_0002189* gene), was successfully developed to quickly distinguish between the vaccine and the causative pathogen strains. In addition, we report the first LAMP trial using comparative genomics designed to distinguish between animals infected with the naturally occurring *Brucella* strains and those that were vaccine immunized. The analysis method is simple and fast, can detect low content DNA in samples, and has high target specificity. It provides an effective detection method for the prevention and control of brucellosis.

## Data availability statement

The datasets presented in this study can be found in online repositories. The name of the repository and accession number can be found below: NCBI; SRP394076.

## Ethics statement

The animal study was reviewed and approved by the Animal Ethics and Welfare Committee of Inner Mongolia Agricultural University. Written informed consent was obtained from the owners for the participation of their animals in this study.

## Author contributions

WW contributed to study design, laboratory supervision, and manuscript editing. CL contributed to study design and manuscript editing. JM contributed to study design, doing experiments, data analysis and manuscript drafting, editing, and writing. QL have contributed equally to this work and share first authorship. XY, XM, YS, and YQ contributed to laboratory quality control and data collection and helped perform the analysis with constructive discussions. All authors contributed to the article and approved the submitted version.

## Funding

This study was supported by Scientific Research Project of Colleges and Universities in Inner Mongolia Autonomous Region (NJZZ19041) and Science and Technology Planning Project of Inner Mongolia Autonomous Region (20140174, 20120244).

## Conflict of interest

The authors declare that the research was conducted in the absence of any commercial or financial relationships that could be construed as a potential conflict of interest.

## Publisher’s note

All claims expressed in this article are solely those of the authors and do not necessarily represent those of their affiliated organizations, or those of the publisher, the editors and the reviewers. Any product that may be evaluated in this article, or claim that may be made by its manufacturer, is not guaranteed or endorsed by the publisher.
